# Extra-Uterine Leiomyoma Presenting After Hysterectomy

**DOI:** 10.7759/cureus.23116

**Published:** 2022-03-13

**Authors:** Chantal Patel, Richard Todd

**Affiliations:** 1 Surgery, University Hospitals of North Midlands, Newcastle-under-Lyme, GBR; 2 Gynecologic Oncology, University Hospitals of North Midlands, Newcastle-under-Lyme, GBR

**Keywords:** extra-uterine, leiomyoma, pelvic mass, letrozole, post-hysterectomy

## Abstract

Leiomyomas are a common gynaecological finding affecting 20-30% of women over the age of 35, with prevalence decreasing following menopause. Around 25% of women present clinically with a leiomyoma, which are most commonly found within the uterus. Rarer extra-uterine locations include the broad ligament, cervix, and vagina. We present a rare case of an extra-uterine leiomyoma located in the perineum of a 59-year-old female. Our case highlights the importance for extra-uterine leiomyoma to be considered as a differential diagnosis in patients presenting with a pelvic mass following hysterectomy.

## Introduction

Leiomyomas are the most common tumour type found within the female pelvis [1]. Around 25% of women present clinically with a leiomyoma, which is most commonly found within the uterus [2]. Rarer extra-uterine locations include the broad ligament, cervix, and vagina [3]. We present a rare case of an extra-uterine leiomyoma located in the perineum of a 59-year-old female.

## Case presentation

An asymptomatic 59-year-old female was referred to the colorectal two-week wait clinic from the community. During gynaecological examination at her general practice, a lump was noted in the peri-anal region. She did not report any weight loss, change in bowel habit, or per rectal bleeding. On examination in clinic, the abdomen was soft and non-tender, on per rectal examination an external mass tracking towards the vagina was palpated. She had a previous surgical history of a total abdominal hysterectomy with left-sided ovarian conservation 15 years previously for multiple uterine leiomyomas. After hysterectomy she had been prescribed oestrogen-only hormone replacement therapy. Differential diagnoses included sarcoma and leiomyoma.

**Figure 1:**
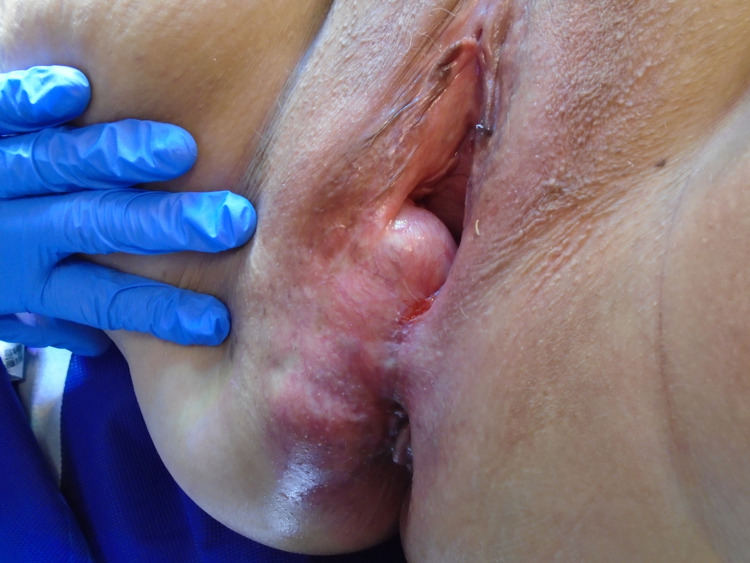
An image of the perineal extra-uterine leiomyoma pre-operatively

**Figure 2:**
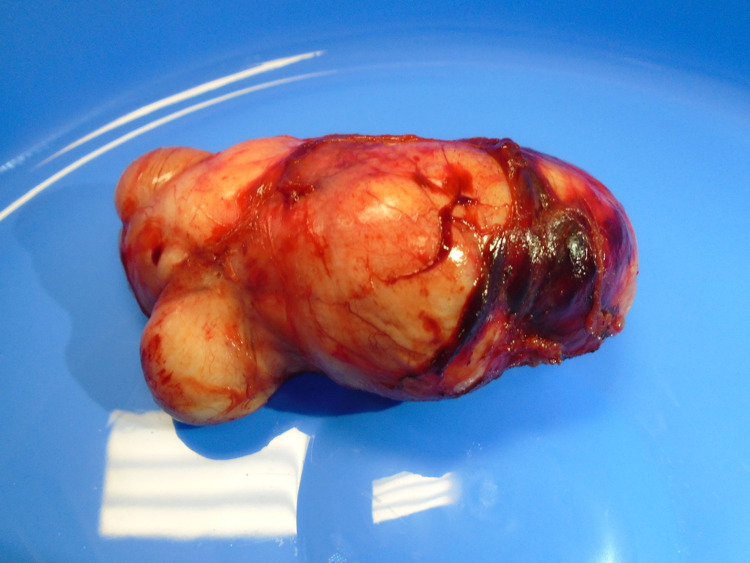
An image of the extra-uterine leiomyoma following excision

Initial biopsy of the mass showed appearances in keeping with a benign leiomyoma. Magnetic resonance imaging revealed a right paramedian perineal mass measuring 4.5 cm × 8.2 cm × 4.2 cm. It bordered the rectum posteriorly, the vagina superior-anteriorly, and the right perineal subcutaneous fat inferiorly. The patient was examined under anaesthesia and commenced on letrozole to reduce the size of the mass pre-operatively. Following multi-disciplinary team discussion, the leiomyoma was excised via a perineal approach (Figures 1, 2). Following the operation, she recovered well and was discharged.

## Discussion

Leiomyomas are a common gynaecological finding affecting 20-30% of women over the age of 35, with prevalence decreasing following menopause [1,3]. Around 25% of women present clinically with a leiomyoma, which is most commonly found within the uterus [2]. Rarer extra-uterine locations include the broad ligament, cervix, and vagina [3]. Uncommon manifestations of leiomyoma include intravenous leiomyomatosis, parasitic leiomyoma, and benign metastasizing leiomyoma [2]. 

Uterine leiomyomas are benign monoclonal tumours of the myometrium consisting of smooth muscle cells, fibroblasts, and extracellular matrix [4,5]. They are encapsulated by a pseudo-capsule formed from areolar tissue and muscle fibres separating the tumour from the local surrounding structures [4]. The growth of leiomyomas is thought to be dependent on vascularization, hormones, and patient age [5].

Common presenting symptoms of uterine leiomyomas include heavy menstrual bleeding, dysmenorrhoea, and pelvic discomfort [1]. They may be discovered following investigation for preterm labour, infertility, and recurrent miscarriage [2]. More unusual presenting symptoms include thrombosis, haematometra, haemoperitoneum, and lower urinary tract symptoms [1]. However, presenting features are dependent on the location of the leiomyoma, in our case no symptoms were noted for the perineal extra-uterine leiomyoma.

Despite often being found incidentally, uterine leiomyomas can be investigated using ultrasonography. Ultrasound provides a fast method in detailing the size, site, and number of uterine fibroids. Magnetic resonance imaging (MRI) is utilized to evaluate the leiomyoma in relation to the rest of the pelvis, including relations to neurovascular structures and nearby organs [3]. The use of MRI as in our case can assist in the planning of operative management. However, imaging methods are not reliable in determining the probability of malignant transformation which is reserved for histological evaluation [3].

Following symptomatic treatment, uterine artery embolization, myomectomy, and hysterectomy are used in the treatment of symptomatic leiomyoma. The patient in our case report had previously undergone a hysterectomy for this indication. Recurrence of leiomyoma, as well as benign metastasizing leiomyoma and leiomyosarcoma after hysterectomy, have been reported in the literature [6,7].

## Conclusions

We present a case of perineal leiomyoma after hysterectomy. Furthermore, our case highlights the importance for extra-uterine leiomyoma to be considered as a differential diagnosis in patients presenting with a pelvic mass after hysterectomy.
